# Expression patterns of immune checkpoints in acute myeloid leukemia

**DOI:** 10.1186/s13045-020-00853-x

**Published:** 2020-04-03

**Authors:** Cunte Chen, Chaofeng Liang, Shunqing Wang, Chi Leong Chio, Yuping Zhang, Chengwu Zeng, Shaohua Chen, Caixia Wang, Yangqiu Li

**Affiliations:** 1 Institute of Hematology, School of Medicine, Key Laboratory for Regenerative Medicine of Ministry of Education, Jinan University, Guangzhou, 510632 People’s Republic of China; 2 Department of Hematology, Guangzhou First People’s Hospital, School of Medicine, South China University of Technology, Guangzhou, 510180 People’s Republic of China

**Keywords:** PD-1, PD-L1, PD-L2, Prognosis, Immune checkpoint, AML

## Abstract

Immunotherapy with immune checkpoint inhibitors (ICIs) for solid tumors had significantly improved overall survival. This type of therapy is still not available for acute myeloid leukemia (AML). One major issue is the lack of knowledge for the expression patterns of immune checkpoints (IC) in AML. In this study, we first explored the prognostic value of ICs for AML patients by analyzing RNA-seq and mutation data from 176 AML patients from the Cancer Genome Atlas (TCGA) database. We further validated the results of the database analysis by analyzing bone marrow (BM) samples from 62 patients with de novo AML. Both TCGA data and validation results indicated that high expression of PD-1, PD-L1, and PD-L2 was associated with poor overall survival (OS) in AML patients. In addition, increased co-expression of PD-1/CTLA-4 or PD-L2/CTLA-4 correlated with poor OS in AML patients (3-year OS: TGCA data 30% vs 0% and 20% vs 0%, validation group 57% vs 31% and 57% vs 33%, respectively) (*P* < 0.05). Moreover, co-expression of PD-1/PD-L1, PD-1/PD-L1/PD-L2, and PD-1/LAG-3 was found to correlate with poor OS in AML patients with FLT3^mut^, RUNX1^mut^, and TET2^mut^, respectively. In conclusion, high expression of ICs in the BM leukemia cells of AML patients correlated with poor outcome. The co-expression patterns of PD-1/CTLA-4, PD-L2/CTLA-4, PD-1/PD-L1, PD-1/PD-L1/PD-L2, and PD-1/LAG-3 might be potential immune biomarkers for designing novel AML therapy.

To the Editor,

Immune checkpoint (IC) blockade by inhibitors of the programmed cell death 1 (PD-1) and PD-1 ligand 1 (PD-L1) has significantly improved clinical outcome for a variety of solid tumors [1, 2], while little is known about the role of ICs in leukemia [3]. Previous reports have shown that higher numbers of PD-1 + T cells are related to poor outcome for patients with acute myeloid leukemia (AML) [3]. Clinical trials using PD-1 inhibitors are ongoing to treat patients with a high risk for AML relapse [4]**.** However, the response rate varies widely, ranging from 22 to 72% [4], which may be due to heterogeneity in the IC expression level as well as distinct dominant IC expression patterns in different AML cases [5]. Therefore, it is worth studying the expression patterns of ICs in AML. In this study, we first explored the prognostic value of ICs in AML patients through analyzing RNA-seq and mutation data from the Cancer Genome Atlas (TCGA) database [6] and further validated the results by quantitative real-time PCR analysis of AML bone marrow (BM) samples from our clinical center.

A total of 176 de novo AML patients from the TCGA database and 62 AML BM samples were used for overall survival (OS) analysis and validation. Higher expression of PD-1, PD-L1, and PD-L2 correlated with poor OS in the TCGA database analysis (3-year OS 23% vs 38%, 19% vs 46%, and 15% vs 40%, respectively, *P* < 0.05). This result was confirmed in the validation group (3-year OS 40% vs 68%, 22% vs 64%, and 42% vs 68%, respectively, *P* < 0.05, Fig. 1a, b). We further analyzed the expression patterns of PD-1, PD-L1, and PD-L2 with other important ICs [7–9]. Subsequently, with Pearson’s correlation analysis, we found that the expression of PD-1, PD-L1, or PD-L2 was positively associated with the expression of cytotoxic T-lymphocyte associated protein 4 (CTLA-4) (*r* = 0.259, *P* < 0.001; *r* = 0.435, *P* < 0.001; *r* = 0.269, *P* < 0.001, respectively) and lymphocyte activation gene-3 (LAG-3) (*r* = 0.275, *P* < 0.001; *r* = 0.276, *P* < 0.001; *r* = 0.160, *P* = 0.033, respectively) in the TCGA group (Fig. 1c). This concomitant expression pattern was again confirmed in the validation group (Fig. 1e), showing the possibility of concomitant expression of PD-1, PD-L1, or PD-L2 with CTLA-4 (*r* = 0.373, *P* = 0.003; *r* = 0.998, *P* < 0.001; *r* = 0.998, *P* < 0.001, respectively) and LAG3 (*r* = 0.372, *P* = 0.003; *r* = 0.994, *P* < 0.001; *r* = 0.994, *P* < 0.001, respectively). AML patients with high expression of CTLA-4 and LAG-3 were found to have poor OS (3-year OS 9% vs 36% and 13% vs 40% respectively) (Fig. 1d). This result was again confirmed in the validation group (Fig. 1f) (3-year OS: CTLA-4 34% vs 66%, LAG-3 33% vs 70%).

**Fig. 1:**
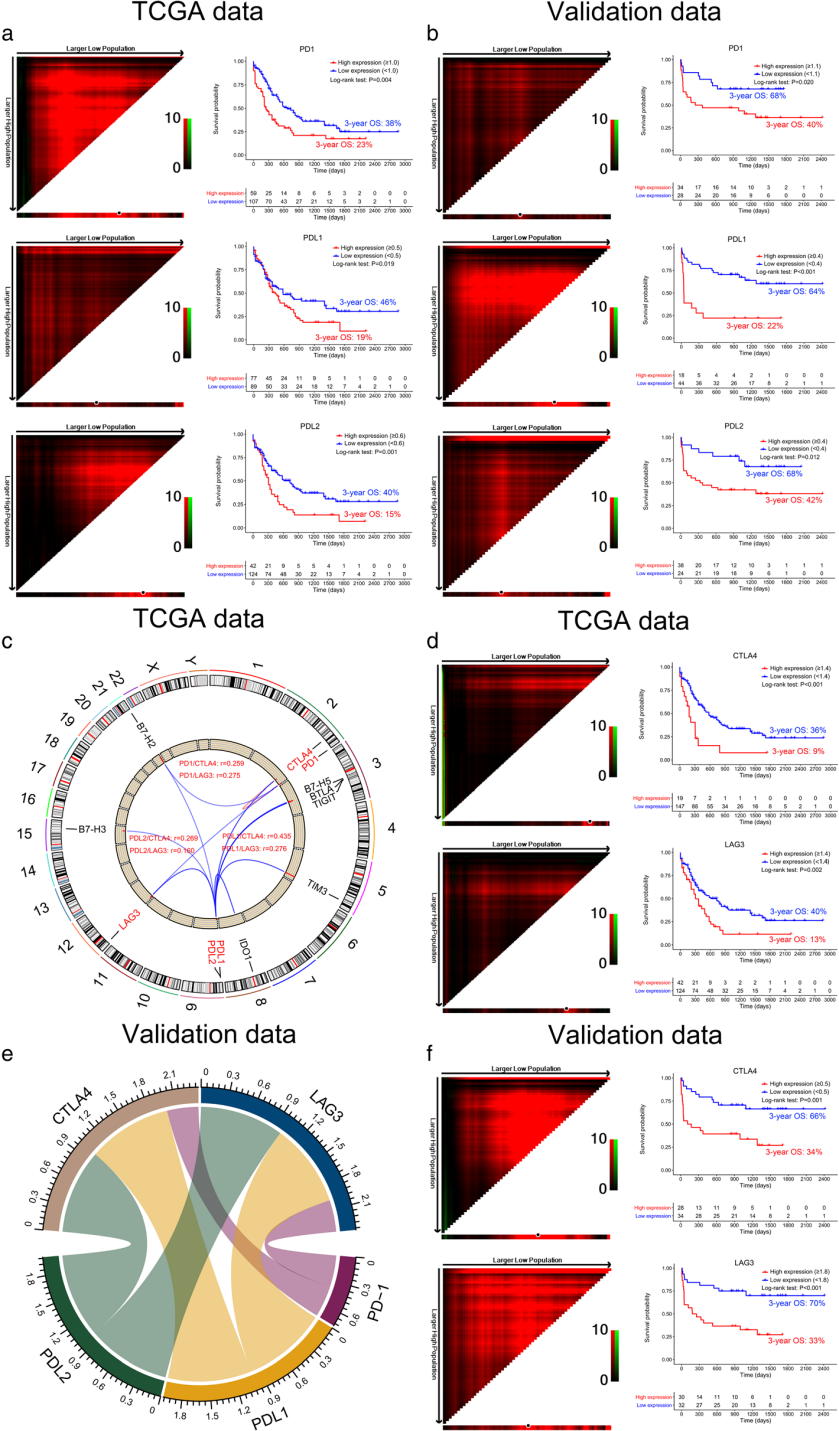
Overall survival (OS) of ICs in AML patients. **a** The OS probability in AML patients with high or low PD-1, PD-L1, or PD-L2 expression in TCGA group. (left panel) X-tile software (version 3.6.1) was used to define the optimal cutoff value for gene expression levels for prognosis, which is represented by the highest intensity pixel. Black dots represent the optimal cutoff value. The black to red or green in the color scale indicates that the range of pixels was from low to high. (right panel) Kaplan–Meier curves based on the optimal cutoff values. **b** The OS probability in AML patients with high or low PD-1, PD-L1, or PD-L2 expression in the validation group (*n* = 62). **c** Relationship between PD-1, PD-L1, and PD-L2 and other immune checkpoints in TCGA group. The outermost circle indicates 1 to 22, X and Y chromosomes; the second layer shows the location of the genes in the chromosomes; the third layer shows the IC genes; the innermost layer represents the average expression levels of the genes, which is shown by the height of the column; the lines in the center of the circle show the co-expression network of the PD-1, PD-L1, and PD-L2 and other ICs. The red font in the center of the circle displays the Pearson’s coefficient with a *P* value < 0.05 for the correlation of two IC genes. **d**, **f** The OS probability in AML patients with high or low CTLA-4 and LAG-3 based on the optimal cutoff values provided by the X-tile software (version 3.6.1) in TCGA group (**d**) and in the validation group (**f**). **e** The chord diagram shows the co-expression network between PD-1, PD-L1, PD-L2, CTLA-4, and LAG-3 in BM samples from AML patients in the validation group (*n* = 62). The band represents a positive correlation between the two IC genes, and the thickness indicates the magnitude of the Pearson’s correlation coefficient (the *P* value for testing the correlation coefficient was < 0.05)

Combination of IC inhibitors (ICIs) has the potential to improve responses [4, 10]. We analyzed expression patterns of ICs and found that pairwise combinations of PD-1, PD-L1, and PD-L2 and CTLA-4 and LAG-3 correlated with poor OS in AML patients (*P* < 0.05, Figure S1). Furthermore, among AML patients with high expressions of PD-1 or PD-L2, concomitant high expression of CTLA-4 correlated with poor OS in both the TCGA database (3-year OS: PD-1^high^CTLA-4^low^ vs PD-1^high^CTLA-4^high^ 30% vs 0%, 20% vs 0%) and validation group (3-year OS: PD-L2^high^CTLA-4^low^ vs PD-L2^high^CTLA-4^high^ 57% vs 31%, 57% vs 33%) (*P* < 0.05, Fig. 2a, b). AML with PD-L1^high^CTLA-4^high^ correlated with poor OS in the TCGA dataset (3-year OS 24% vs 0%, *P* < 0.001); however, OS was not significantly different in the validation group (3-year OS 33% vs 20%, *P* = 0.353, Fig. 2a, b). In addition, high expression of LAG-3 with PD-1^high^, PD-L1^high^, or PD-L2^high^ failed to correlate with OS in the TCGA and validation groups (Figures S2A - B).

**Fig. 2:**
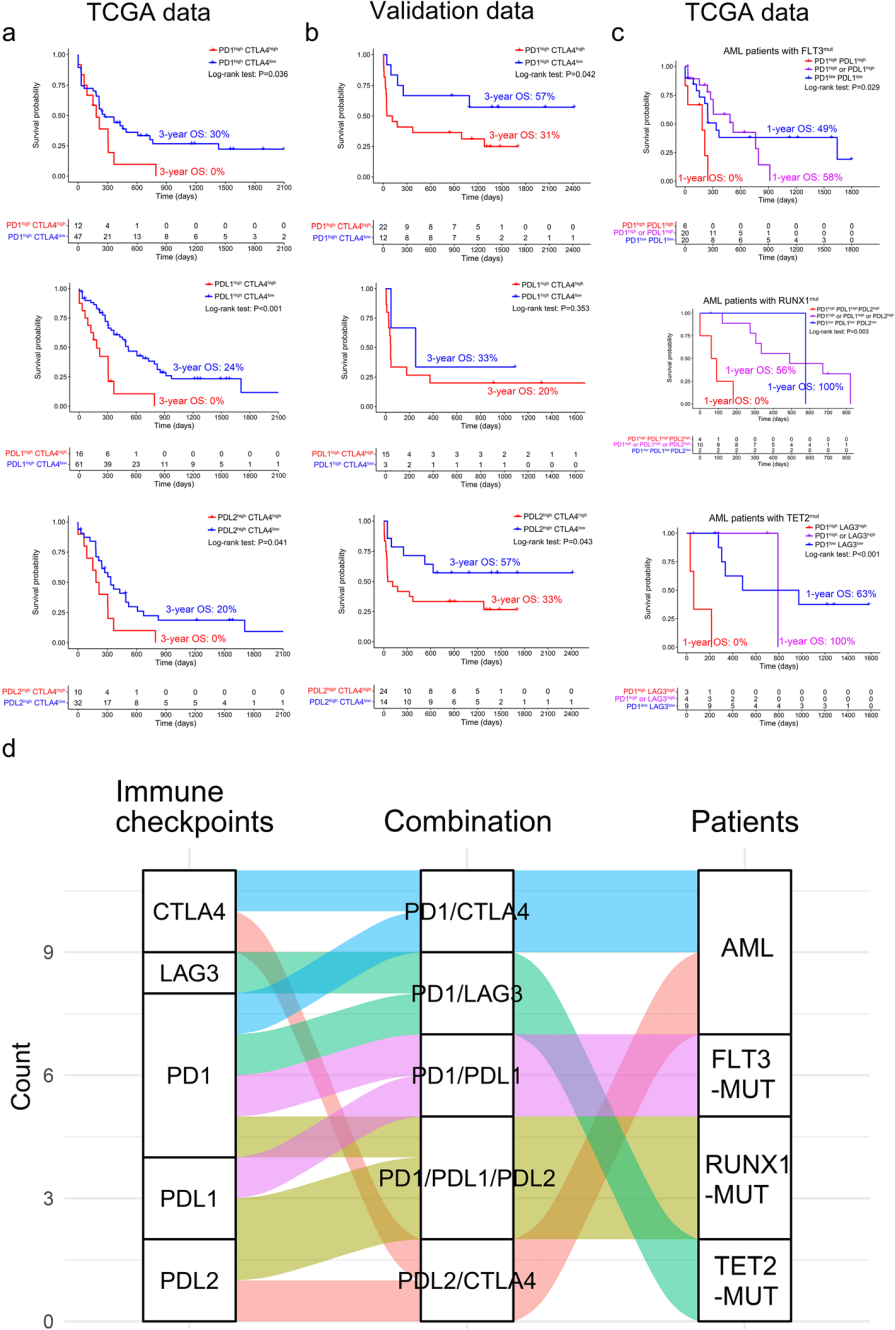
Co-expression patterns of ICs related to poor OS in AML patients. **a**, **b** Comparison of OS curves in AML patients with PD-1^high^, PD-L1^high^, or PD-L2^high^ co-expressed with CTLA-4^low^ or CTLA-4^high^ in TCGA group (**a**) and the validation group (*n* = 62) (**b**), respectively. **c** Comparison of OS curves in AML patients with or without FLT3, RUNX1, or TET2 mutation in TCGA group. mut mutation, wt wildtype. **d** Schematic summary of optimal IC combination detection for OS analysis in AML patients with genetic mutations

To obtain the effects of PD-1, PD-L1, and PD-L2 on the prognosis of AML patients with genetic mutations, we analyzed OS of the top ten AML patients with a recurrent mutation (Figures S3), including FLT3^mut^, RUNX1^mut^, or TET2^mut^ [11–13]. Interestingly, in comparison with AML patients without such mutations, high co-expressions of PD-1/PD-L1 (*P* = 0.029), PD-1/PD-L1/PD-L2 (*P* = 0.003), and PD-1/LAG-3 (*P* < 0.001) were found to be associated with poor OS in AML patients with FLT3^mut^, RUNX1^mut^, or TET2^mut^ (1-year OS 0% vs 58% vs 49%, 0% vs 56% vs 100%, and 0% vs100% vs 63%, respectively) (Fig. 2c).

To the best of our knowledge, we for the first time described that high co-expressions of PD-1/CTLA-4 and PD-L2/CTLA-4 correlated with poor OS of AML patients. Moreover, high co-expressions of PD-1/PD-L1, PD-1/PD-L1/PD-L2, and PD-1/LAG-3 were associated with poor OS of AML patients with FLT3^mut^, RUNX1^mut^, or TET2^mut^, respectively (Fig. 2d). These co-expression patterns might be potential immune biomarkers for designing novel AML therapy.

## Supplementary information

**Additional file 1: Figure S1.** Combination of IC detection for OS analysis in patients with AML. A and B: Comparison of OS in patients with high or low expression of PD-1, PD-L1, or PD-L2 co-expressed with high or low CTLA-4 or LAG-3 in the TCGA group (A) and in the validation group (n = 62) (B), respectively.

**Additional file 2: Figure S2.** Comparison of OS in AML patients with high expression PD-1, PD-L1 or PD-L2 co-expressed with high or low LAG3 in the TCGA group (A) and the validation group (B).

**Additional file 3: Figure S3.** Mutation landscape of the top 10 genes in 176 AML patients in the TCGA database.

**Additional file 4: Table S1.** Clinical information for the AML patients.

**Additional file 5: Table S2.** The primers for qRT-PCR.

**Additional file 6: Materials and Method**

## Data Availability

All supporting data are included in the manuscript and supplemental files. Additional data are available upon reasonable request to the corresponding author.

## Abbreviations

AML: Acute myeloid leukemia
BM: Bone marrow
CTLA-4: Cytotoxic T-lymphocyte associated protein 4
FLT3: Fms-related receptor tyrosine kinase 3
IC: Immune checkpoint
ICI: Immune checkpoint inhibitor
LAG-3: Lymphocyte activation gene-3
mut: Mutation
PD-1: Programmed cell death 1
PD-L1: Programmed cell death 1 ligand 1
PD-L2: Programmed cell death 1 ligand 2
RUNX1: RUNX family transcription factor 1
TCGA: The Cancer Genome Atlas
TET2: Tetmethylcytosine dioxygenase 2
wt: Wildtype
