# Supplementary figures and images for: Expression patterns of immune checkpoints in acute myeloid leukemia
Source: J Hematol Oncol. 2020 Apr 3;13:28. doi: 10.1186/s13045-020-00853-x (PMC7118887; doi:10.1186/s13045-020-00853-x)

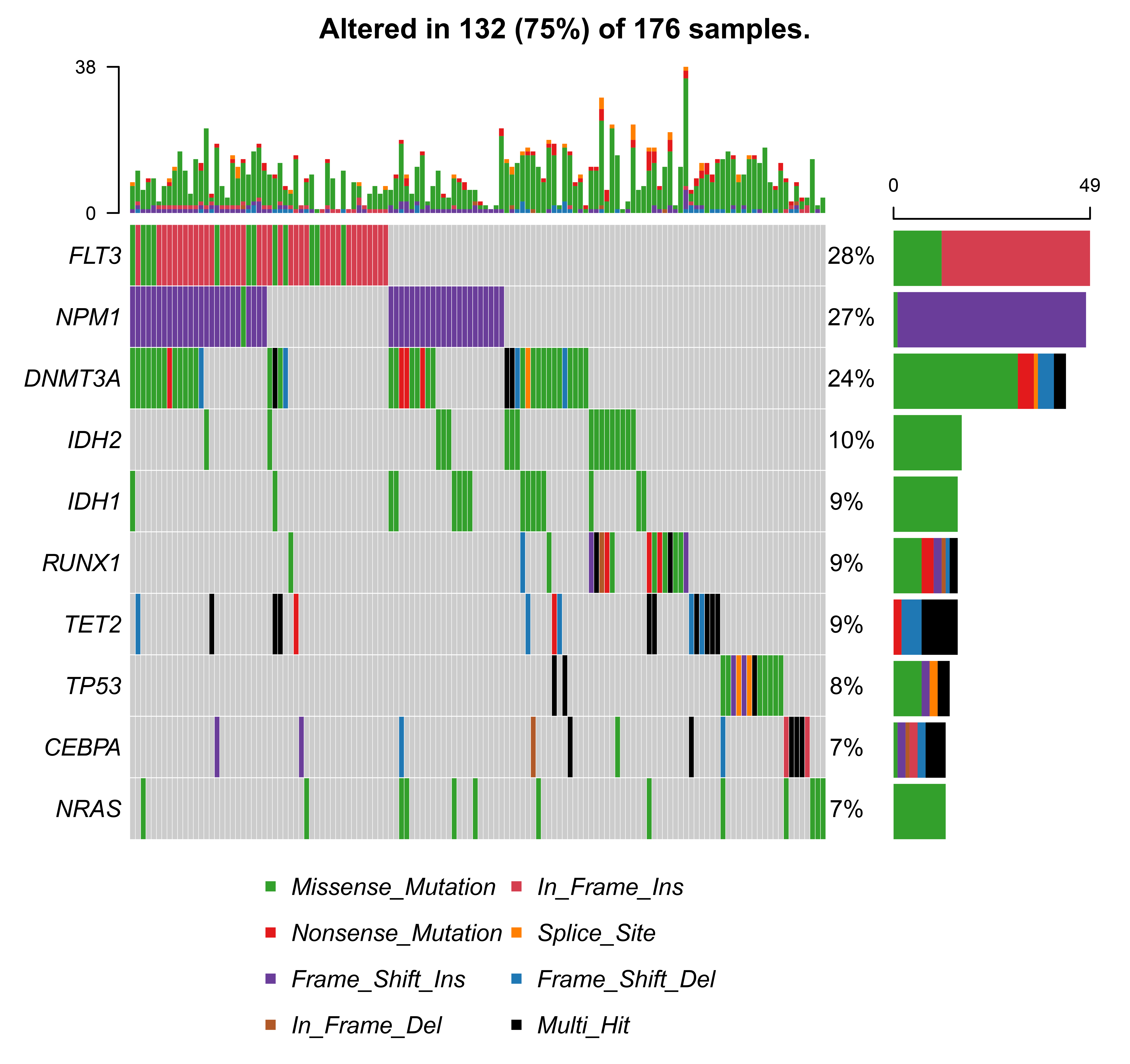

Supplement: Supplementary file 3 — Additional file 3: Figure S3. Mutation landscape of the top 10 genes in 176 AML patients in the TCGA database. [file 13045_2020_853_MOESM3_ESM.tif]
