# Supplementary material for: Expression patterns of immune checkpoints in acute myeloid leukemia
Source: J Hematol Oncol. 2020 Apr 3;13:28. doi: 10.1186/s13045-020-00853-x (PMC7118887; doi:10.1186/s13045-020-00853-x)
Supplement: Supplementary file 4 — Additional file 4: Table S1. Clinical information for the AML patients. [file 13045_2020_853_MOESM4_ESM.docx]

**Table S1.** Clinical information for the AML patients.

| **Variables** | **Total** | **Training group** | **Validation group** | **P-value** |
| --- | --- | --- | --- | --- |
| Number | 238 | 176 | 62 |  |
| Age, mean±SD, years |  | 55±16 | 43±17 | <0.001^***^ |
| Gender, n (%) |  |  |  | 0.758 |
| Female | 115(48.3) | 84 (47.7) | 31 (50.0) |  |
| Male | 123(51.7) | 92 (52.3) | 31 (50.0) |  |
| Risk stratification, n (%) |  |  |  | <0.001^***^ |
| Low | 45(18.9) | 32 (18.2) | 13(21.0) |  |
| Intermediate | 134(56.3) | 103 (58.5) | 31(50.0) |  |
| High | 46(19.3) | 39 (22.2) | 7(11.3) |  |
| Unknown | 13(5.5) | 2 (1.1) | 11(17.7) |  |
| Cytogenetic abnormality, n (%) |  |  |  | 0.020^*^ |
| No | 115(48.3) | 85 (48.3) | 30(48.4) |  |
| Yes | 95(39.9) | 76 (43.2) | 19(30.6) |  |
| Unknown | 28(11.8) | 15 (8.5) | 13(21.0) |  |
| Subtype, n (%) |  |  |  | <0.001^***^ |
| M0 | 15(6.3) | 15 (8.5) | 0(0.0) |  |
| M1 | 43(18.1) | 42 (23.9) | 1(1.6) |  |
| M2 | 60(25.2) | 41 (23.3) | 19(30.6) |  |
| M3 | 24(10.1) | 16 (9.1) | 8(12.9) |  |
| M4 | 39(16.4) | 34 (19.3) | 5(8.1) |  |
| M5 | 43(18.1) | 21 (11.9) | 22(35.5) |  |
| M6 | 7(2.9) | 2 (1.1) | 5(8.1) |  |
| M7 | 3(1.3) | 3 (1.7) | 0(0.0) |  |
| Unclassified | 7(2.9) | 2 (1.1) | 5(8.1) |  |

*, P<0.05; ***, P<0.001.
