# Supplementary material for: Expression patterns of immune checkpoints in acute myeloid leukemia
Source: J Hematol Oncol. 2020 Apr 3;13:28. doi: 10.1186/s13045-020-00853-x (PMC7118887; doi:10.1186/s13045-020-00853-x)
Supplement: Supplementary file 5 — Additional file 5: Table S2. The primers for qRT-PCR. [file 13045_2020_853_MOESM5_ESM.docx]

**Table S2.** The primers for qRT-PCR.

| Target | Sequence 5' - 3' |
| --- | --- |
| PD-1 (F) | CTCAGGGTGACAGAGAGAAG |
| PD-1 (R) | GACACCAACCACCAGGGTTT |
| PD-L1 (F) | TATGGTGGTGCCGACTACAA |
| PD-L1 (R) | TGCTTGTCCAGATGACTTCG |
| PD-L2 (F) | ACAGTGCTATCTGAACCTGTGG |
| PD-L2 (R) | CTGCAGGCCACCGAATTCTT |
| CTLA-4 (F) | GCCCTGCACTCTCCTGTTTTT |
| CTLA-4 (R) | GGTTGCCGCACAGACTTCA |
| LAG-3 (F) | CTAGCCCAGGTGCCCAACGC |
| LAG-3 (R) | GCCTGCGGAGGGTGAATCCC |
| 18SrRNA (F) | CGGCGGCTTTGGTGACTCTAGA |
| 18SrRNA (R) | CCTGCTGCCTTCCTTGGATGTG |
