# Supplementary material for: Expression patterns of immune checkpoints in acute myeloid leukemia
Source: J Hematol Oncol. 2020 Apr 3;13:28. doi: 10.1186/s13045-020-00853-x (PMC7118887; doi:10.1186/s13045-020-00853-x)
Supplement: Supplementary file 6 — Additional file 6: Materials and Method [file 13045_2020_853_MOESM6_ESM.docx]

**Materials and Methods**

**Acquisition of the TCGA dataset**

Level 3 RNA-seq data from 179 AML patients, and mutation data from 197 AML patients together with corresponding clinical information were downloaded from the TCGA (<https://cancergenome.nih.gov/>) database using the UCSC XENA (<https://xenabrowser.net/datapages/>). RNA-seq data are presented in the form of log2 (RPKM + 1), and the type of mutation data included somatic mutations (SNP and INDEL). Finally, there were 176 AML patients with both RNA-seq and mutation data who were used for subsequent analyses. The clinical information used for analysis included age, gender, overall survival time, status, subtype, risk stratification, and cytogenetics. RNA-seq data from 176 of the AML patients were assigned to a training group for analysis, and their clinical information is listed in **Table S1**. Because the TCGA dataset is publicly available, no local ethics committee approval was required.

**AML patient samples**

During the period ranging from January 1, 2013 to December 31, 2018, a total of 62 BM samples were obtained from patients newly diagnosed with AML at the Guangzhou First People's Hospital and were assigned to the validation group. Clinical information from 62 patients was also collected, including age, gender, overall survival time, status, subtype, risk stratification, and cytogenetics (**Table S1)**. The last follow-up time was on December 31, 2019, and the median follow-up time for the 62 surviving patients was 1,368 days (range: 682-2,416 days). In addition, BM samples from 12 healthy individuals were used as controls. This study was approved by the Ethics Committee of Guangzhou First People's Hospital. All participants provided written informed consent.

### Extraction of RNA and quantitative real-time PCR (qRT-PCR)

Total RNA was extracted based on the manufacturer's protocol for the TRIzol reagent (Invitrogen). RNA was reverse transcribed into cDNA using a reverse transcription kit (Promega, USA) [1, 2]. The expression levels of PD-1, PD-L1, PD-L2, CTLA-4, and LAG-3 were quantified with a qRT-PCR kit (TIANGEN, China), and 18S rRNA was used as an internal control using the Real-Time System (Bio-Rad, USA) [3]. The list of primers for qRT-PCR are shown in **Table S2**. The expression levels of PD-1, PD-L1, PD-L2, CTLA-4, and LAG-3 are presented as 2^-ΔΔCT^ values.

**Obtaining an optimal cutoff value by X-tile**

X-tile software (version 3.6.1) [4] is a bioinformatics tool that is primarily used to determine the optimal prognostic threshold for quantitative data. X-tile software is currently widely used to analyze the prognosis of cancer patients [2, 5]. X-tile plots were drawn by dividing the gene expression levels into two groups: low and high. Survival curves were plotted for all possible gene expression cutoff values and evaluated by the log-rank test. Finally, the cutoff value with the largest χ^2^ and smallest P value was determined as the optimal cutoff value for gene expression.

**Statistical analysis**

All statistical analyses were performed using SPSS (version 16.0, Inc, Chicago, IL, USA) and GraphPad Prism (version 5.0, CA, USA) as appropriate. Differences between two groups of quantitative data were determined by the Student's t test. Categorical variables were compared by the Chi-square test. Differences in Kaplan-Meier curves were analyzed by the log-rank test. Correlations in the expression levels of two ICs are presented with Pearson coefficients. A two-tailed P value <0.05 was considered statistically significant.

**References**

1. Chen C, Wang P, Mo W, Zhang Y, Zhou W, Deng T, et al. lncRNA-CCDC26, as a novel biomarker, predicts prognosis in acute myeloid leukemia. Oncol Lett. 2019; doi:10.3892/ol.2019.10591.

2. Chen CT, Wang PP, Mo WJ, Zhang YP, Zhou W, Deng TF, et al. Expression profile analysis of prognostic long non-coding RNA in adult acute myeloid leukemia by weighted gene co-expression network analysis (WGCNA). Journal of Cancer. 2019; doi:10.7150/jca.31234.

3. Chengwu Zeng, Sichu Liu, Shuai Lu, Xibao Yu, Jing Lai, Yifan Wu, et al. The c-Myc-regulated lncRNA NEAT1 and paraspeckles modulate imatinib-induced apoptosis in CML cells. Mol Cancer. 2018; doi:10.1186/s12943-018-0884-z.

4. Camp RL, Dolled-Filhart M, Rimm DL. X-tile: a new bio-informatics tool for biomarker assessment and outcome-based cut-point optimization. Clin Cancer Res. 2004; doi:10.1158/1078-0432.CCR-04-0713.

5. Chen C, Wang P, Wang C. Prognostic nomogram for adult patients with acute myeloid leukemia: A SEER database analysis. Medicine (Baltimore). 2019; doi:10.1097/MD.0000000000015804.
